# Understaffed Home Nursing and Wellbeing of Families of Children with Medical Complexity

**DOI:** 10.3390/children12040455

**Published:** 2025-03-31

**Authors:** S. Margaret Wright, Brian Lee, Leslee T. Belzer, Emily J. Goodwin, Jeffrey D. Colvin

**Affiliations:** 1 Division of General Academic Pediatrics, Children’s Mercy Kansas City, Kansas City, MO 64111, USA; ejgoodwin@cmh.edu (E.J.G.); jdcolvin@cmh.edu (J.D.C.); 2 Department of Pediatrics, School of Medicine, University of Missouri-Kansas City, Kansas City, MO 64108, USA; blee@cmh.edu (B.L.); ltbelzer@cmh.edu (L.T.B.); 3 School of Medicine, University of Kansas, Kansas City, KS 66160, USA; 4 Office of Health Services and Outcomes Research, Children’s Mercy Research Institute, Children’s Mercy Kansas City, Kansas City, MO 64108, USA; 5 Division of Developmental and Behavioral Health, Section of Pediatric Psychology, Children’s Mercy Kansas City, Kansas City, MO 64108, USA

**Keywords:** home nursing, care access, family wellbeing, children with medical complexity, pediatric home healthcare, acute care rate, financial hardship

## Abstract

**Background/Objectives**: Some children with medical complexity (CMC) require home nursing (HN) to maintain their health, but many families have difficulty staffing approved HN hours. Little is known about the relationship between understaffed HN, the acute care encounter rate, and family wellbeing. This study examined the association between understaffed HN, acute care encounters, and family wellbeing among CMC. **Methods**: We completed a cross-sectional survey study of caregivers of CMC age 0–17 years at a children’s hospital in the Midwest US. The primary predictors were the proportion of staffed to approved HN hours and the acute care rate. The primary outcome was family wellbeing, measured as parental stress, family impact, interpersonal support, parental empowerment, and financial hardship. General linear models were used to model continuous family wellbeing outcomes. Poisson models were used to calculate the financial hardship summed score ([0–4]). **Results**: Receipt of <50% of approved HN hours was associated with family financial hardship in adjusted analyses. Total family impact and health-related quality of life (HRQL) scores were associated with the acute care rate, with more positive scores among CMC within the middle tertile for the acute care rate compared to the lowest tertile. There was no association between HN staffing and family wellbeing, or between acute care rate and family financial hardship. **Conclusions**: High levels of financial hardship were significantly associated with understaffed HN hours. Family impact and parental HRQL were associated with the acute care rate. Access to HN services carries potential family-level and system-level benefits for this complex and high acuity pediatric population.

## 1. Introduction

Children with medical complexity (CMC) have complex chronic conditions, often associated with functional impairment, medical technology dependence, and increased healthcare utilization [1]. While only about 5% of children are CMC, they account for one fourth of pediatric acute care hospitalizations and approximately 40% of pediatric inpatient costs in the US [2]. CMC are more likely than other children to require emergency department (ED) care and have increased odds of revisiting the ED and subsequent hospitalization [3,4]. While much research has examined CMC’s readiness for a safe discharge from the hospital [5,6], little is known about promoting and maintaining health and wellbeing in the home setting [7,8]. Despite frequent healthcare encounters, most care for CMC is provided at home [9]. Due to their extensive healthcare needs, home nursing (HN) is an integral part of care for some CMC. HN is a home healthcare service in which a registered nurse or licensed practical nurse provides clinical services in the home for extended periods [10]. Without this care, some CMC could not live safely at home [11,12,13]. Within the US, HN services are guided by federal law but are administered by states through Medicaid’s joint federal–state partnership. Eligibility determinations and allocation of HN hours are performed by state Medicaid fee-for-service, managed care organizations, or both, depending on the state [14,15,16]. In the US, a combination of high demand and a fragmented, poorly compensated workforce have contributed to a national HN shortage [17,18,19,20]. Internationally, CMC face similar challenges regarding access to home healthcare services [21,22,23,24]. As a result, many CMC who qualify for HN are unable to obtain it.

Parents (in this study, the term “parent” is inclusive of any legal guardian who serves as a primary caregiver for CMC) of CMC experience high caregiving demands and unmet caregiver needs [25,26]. Hospitalizations bring stress and family disruptions that have a negative impact on both parent and CMC wellbeing [27]. The psychosocial stress of caregiving for CMC has been well described [28,29,30]. Parents of CMC report lower wellbeing, mental health, and sleep-related health compared to US population norms [31]. Financial and social hardships are common among families of CMC [32,33,34,35]. Out-of-pocket costs and financial distress are more common among children and adults with chronic conditions [36,37]. Parents of CMC have described the challenges of balancing employment with unpredictable caregiving needs [38]. Similarly, forgone family employment is associated with increased complexity among the broader population of children and youth with special healthcare needs [39,40].

Prior research on pediatric home healthcare has described a “leaky pipeline” in which “leaks” or gaps in HN policies and programs lead to reduced HN access and increased unpaid care by parents [41]. Gaps in insurance access and variable eligibility determinations are followed by difficulty assigning nurses to staff hours and inconsistent nurse shift staffing. In qualitative studies, parents of CMC have reported the negative impact of understaffed HN on marital and family dynamics, parental stress, parental employment, and out-of-pocket costs [5,17,42,43]. Importantly, during times of adequate HN staffing, parents reported that improved symptom control and chronic disease management led to CMC’s ability to participate in family and school activities [44]. In light of fragmented HN delivery systems that require complex data collection, prior quantitative studies have generally been limited to describing the impact of HN access on acute care utilization, and most describe the number of HN hours approved or received, but not both [20]. An exception is a small cross-sectional survey study of children receiving palliative care which described an average gap between approved and staffed HN of 40 h/week, which parents associated with hospital discharge delays, missed work, and about 10 h/month spent searching for HN staffing [45]. The impact of the widespread reality of understaffed HN on child health and family wellbeing is not fully understood. Our objective was to examine the association between understaffed HN in CMC, acute care encounters, and measures of family wellbeing.

## 2. Materials and Methods

We conducted a cross-sectional survey study of parents of CMC receiving primary care within a large children’s hospital system in the Midwest US. The parents were participants in a CMC research repository; informed consent was obtained from all participants at the time of repository enrollment. CMC were age 0–17 and receiving primary care within a dedicated clinic for CMC within the hospital system during the 5-year study period (2015–2019). We elected to conclude the study period prior to the onset of the SARS CoV-2 pandemic to avoid the effects confounding on HN staffing. The primary care clinic dedicated to CMC routinely collects and records detailed documentation regarding HN staffing on an ongoing basis. The inclusion criteria also required all participants to have approved HN services for at least thirty days immediately prior to completion of the parent survey. This study was approved by the hospital’s institutional review board.

### 2.1. Data Sources

Approved and staffed HN hours were extracted from the electronic health record (EHR) for each participant who met the inclusion criteria. Centers for Medicare & Medicaid Services 485 Forms (Home Health Plan of Care) served as the gold standard for documentation of approved HN hours. This form provided documentation of insurance-approved HN hours and was stored in the EHR as it required physician signature every 60 days. The EHR was searched for key terms to identify documentation of fulfillment of home nursing services using a natural language processing (NLP) approach. The NLP terms used in this study have been taken from previously published HN research [46] and reviewed and modified for completeness. The investigation team has previously successfully developed NLP algorithms for manipulating and extracting text from provider notes. We performed a pilot extraction of HN data using natural language and validated the process through manual chart reviews prior to initiating the data query for this study. We developed a priori standardized assumptions for the interpretation of non-numeric descriptions of HN hour staffing (Supplementary Table S1). Acute encounter and demographic data were collected from the Pediatric Health Information System (PHIS), an administrative database of 50 tertiary children’s hospitals in the US. Outcomes related to family wellbeing were collected from surveys administered to the parents.

### 2.2. Main Predictor: Staffed Home Nursing

Staffed HN for this study was defined as the weekly number of HN hours staffed divided by the number of hours approved per week. Our main predictor was the median percentage of staffed HN over the 30 days prior to survey completion. This allowed us to identify associations with understaffed HN, rather than the discrete number of HN hours approved, as our objective was to assess HN service delivery. We created three categories based on the median percentage of staffed HN: <50%, 50–89%, and ≥90%. The highest category (90–100%) was based on the rulings of federal litigation related to understaffed HN in the state of Florida in 2023. In that ruling, 90% was determined to be the minimum necessary percentage of staffed HN [47]. We dichotomized all HN staffing <90% into two equal groups based on frequency.

### 2.3. Secondary Predictor: Acute Care Rate

We measured the acute care rate as our secondary predictor. We defined the acute care rate as the number of acute care encounters (emergency department visits or hospital admissions) up to 180 days prior to completion of parent survey and then standardized per days of approved HN.

### 2.4. Main Outcome

The main outcome was family wellbeing. As family wellbeing has many facets, we operationalized family wellbeing as parental stress, parental health-related quality of life (HRQL), family functioning, interpersonal support, family empowerment, and financial hardship.

- Parental Stress: We measured parental stress using Cohen’s Perceived Stress Scale. This validated 10-item scale is frequently used in the health literature to measure patient or caregiver stress. It is a continuous scale with a higher score being indicative of more stress [48].
- Parental HRQL and Family Functioning: We used the 36-item PedsQL Family Impact Module (PedsQL-FIM) to measure parental HRQL and family functioning. The PedsQL-FIM results in a total score as well as individual scores for the two subscales, parental HRQL and family functioning. The parental HRQL subscale is measured through 20 items assessing self-reported physical, emotional, social, and cognitive functioning, communication, and worry. The 8-item Family Functioning subscale measures family daily activities and family relationships. Lower scores indicate a greater impact of the child’s condition on the family overall (total score) and the parental HRQL and family functioning sub-scales. It has been validated in both CMC and a community sample of children [49,50].
- Parental Interpersonal Support: We used the 12-item Interpersonal Support Evaluation List (ISEL-12) to measure parental interpersonal support. The ISEL-12 is a validated measure of functional social support including the constructs of appraisal, belonging, and tangible social support. Scores are along a continuous scale, with higher values indicating greater social support [51,52].
- Family Empowerment: We used the Family Empowerment Scale (FES), which is a validated instrument for measuring empowerment in parents of children with chronic health problems. The FES includes the constructs of empowerment related to family, service systems, and community/political systems. FES scores are along a continuous scale, with higher values indicating greater empowerment [53,54,55].
- Family Financial Hardship: Family financial hardship was measured using validated survey questions relating to household material hardship (food, housing, and energy insecurity) and forgone employment (FFE), defined as a caregiver who reduced work hours or left work due to their child’s health. The four binary indicators for material hardship and FFE were summed to create a composite score. Assessment of household material hardship in these three domains has been previously validated by Children’s HealthWatch, a network of public health researchers and child health and policy experts studying the health consequences of family economic hardship [56,57]. Validated survey questions regarding FFE were derived from the National Survey of Children’s Health, a nationally representative survey of child health and family interactions funded and directed by the Maternal and Child Health Bureau [58].

### 2.5. Covariates

Our covariates included patient age, sex, insurance type, self-identified race/ethnicity, and number of complex chronic conditions (CCCs). We included the social construct of race/ethnicity to identify possible inequities with receipt of HN services. These data were collected from PHIS. Insurance type was collected from all PHIS encounters during the study period and reflects the payor for acute care encounters, with one payor per encounter. Participants with both public and commercial insurance had encounters that were paid for by both public and commercial payors. CCCs were identified using Feudtner’s CCC Classification System [59]. We included approved HN hours per week, standardized to 8 h shifts as a proxy for a child’s complexity and acuity.

### 2.6. Analytic Approach

The first complete survey response following at least 30 days of HN eligibility was included for each participant. Continuous family wellbeing outcome variables were standardized (i.e., mean 0, standard deviation 1) to allow for meaningful interpretation and for comparability between wellbeing metrics; variables that exhibited skewness were transformed prior to standardization. Due to data skewness, the acute care rate was divided into tertiles rather than treated as a continuous variable. General linear models were used to model continuous family wellbeing outcomes, while Poisson models were used for the financial hardship summed score ([0–4]). Adjusted models included HN staffing category, categorized acute care rate, number of CCCs (i.e., 2–3, 4–5, ≥6), and participant age in years at the initiation of HN (i.e., <1, 1–2, 3–5, 6–11, and 12–17). Bootstrapped confidence intervals are provided for all regression coefficients and are based on 2000 replications. All analyses were completed using R software, version 4.3.3 (R Core Team; Vienna, Austria) and Stata (*Stata Statistical Software: Release 18.5*, College Station, TX, USA).

## 3. Results

### 3.1. Participants

The study population’s characteristics are displayed in Table 1. Seventy-nine percent of participants had more than three CCCs. Fifty-eight percent of participants were publicly insured. Eighty-two percent had insurance approval for more than 40 h of HN per week. Thirty-nine percent of participants had less than 90% of their approved HN hours staffed.

### 3.2. Association of HN Staffing with Family Wellbeing

**Figure 1:**
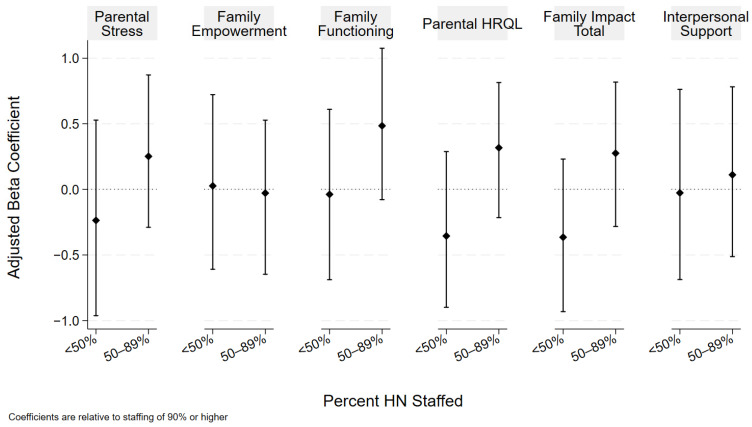
HN staffing and family wellbeing. Coefficients and bootstrapped confidence intervals are relative to the referent of ≥90% HN. Analyses adjusted for age, number of CCCs, and acute care rate.

There were no observed associations between the proportion of staffed HN and parental stress, interpersonal support, empowerment, or the PedsQL-FIM total score or its subscale scores for parental HRQOL and family functioning. Figure 1 displays our results, adjusted for age, number of CCCs, and the acute care rate. The results of both the unadjusted and adjusted analyses are included in Table 2.

Eighty-nine percent of participants reported at least one type of family financial hardship (Supplementary Table S2). FFE was common and was reported by 83.3% of all participants and by 93.8% of those reporting any hardship. Receipt of less than 50% of approved HN hours (compared to receipt of ≥90% of HN) was associated with a 50% higher adjusted family financial hardship rate, as measured using a composite count of forgone family employment, food insecurity, housing insecurity, and energy insecurity (adjusted rate ratio 1.51 (CI 1.03, 2.16) Table 3).

### 3.3. Association of Acute Care Utilization and Family Wellbeing

**Figure 2:**
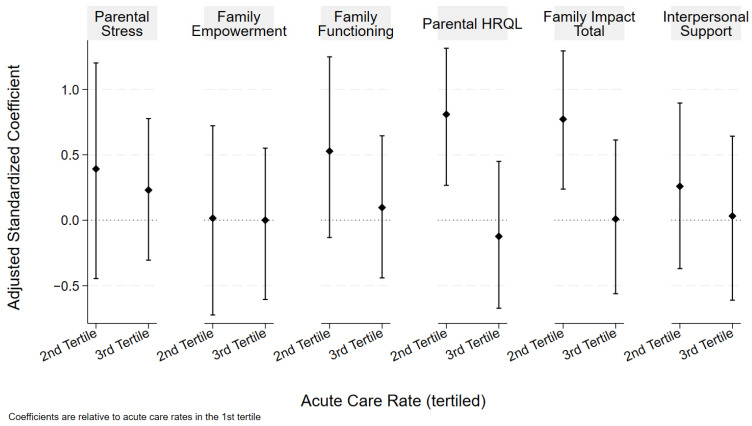
Acute care rate and family wellbeing. Coefficients and bootstrapped confidence intervals are relative to referent of first tertile (lowest acute care rate). Analyses adjusted for age, number of CCCs, and percentage of HN staffing.

Acute care utilization was associated with the PedsQL-FIM total score and subscale score for parental HRQOL (Figure 2). Participants within the middle tertile of the acute care rate (compared to those in the lowest acute care tertile) had higher adjusted total PedsQL-FIM total scores for family impact (adjusted beta coefficient 0.77 (CL 0.24, 1.30)) and a higher parent-reported health-related quality of life (FIM HRQL adjusted beta coefficient 0.81 (CL 0.27, 1.32)). The results of both the unadjusted and adjusted analyses are included in Table 4. There were no significant associations between acute care utilization and the family functioning subscale of the PedsQL-FIM or parental stress, interpersonal support, or empowerment. There was also no significant association between acute care utilization and the presence of family financial hardship.

## 4. Discussion

This cross-sectional survey study examined the association between access to approved pediatric home nursing, acute care encounters, and family wellbeing among CMC. Family wellbeing is a multifaceted construct, closely related to quality of life and influenced by social, community, financial, physical, and psychosocial factors [60,61,62]. In part because of this conceptual complexity, there is no universal gold standard for its measurement [63]. In the pediatric healthcare literature, this concept is often measured and described as health-related quality of life or reduced to certain dimensions such as caregiver mental health or sleep quality [64]. With parent and family wellbeing linked to caregiving capacity and child health [65,66], it is essential to understand the ways in which HN access may affect wellbeing for the families of CMC. In this study, receipt of less than half of a CMC’s approved HN hours was associated with a fifty percent increase in the family financial hardship rate. This association persisted after adjusting for age, number of CCCs, and the acute care rate. Participants within the middle tertile of the acute care rate had more positive scores in measures of family impact (PedsQL FIM total score) and parental health-related quality of life compared to those in the lowest acute care tertile. There were no significant associations between HN staffing and other measures of family wellbeing. There was also no association between the acute care rate and the presence of family financial hardship.

We found a significant association between receipt of less than 50% of approved HN hours and the presence of family financial hardship. This finding includes both forgone employment itself and the financial hardships (food, housing, and energy insecurity) which can accompany income loss. A lack of full receipt of approved HN shifts the caregiving role to families [8], a known factor in financial hardship that is inversely related to family wellbeing [67,68,69,70]. Parents of CMC have described the challenges in balancing the need to work to support their families with the need for flexibility to care for a child with complex and at times unpredictable healthcare needs [38,71]. This finding resonates with what we hear from families clinically: when they cannot staff enough HN hours, parents manage that medical care themselves, at the cost of employment and other responsibilities. The high frequency of reported financial hardship and the high proportion of families with forgone employment due to their CMC’s health in this study are also notable. Whereas approximately 15% of families of children with special healthcare needs report forgone employment [39], over 80% of participants in the present study cut down on work hours or left work due to their CMC’s health (Supplementary Table S2). CMC are a complex subset of children with special healthcare needs, and the need for HN services may be a factor impacting these families’ ability to maintain work. In several US states, policies supporting paid family caregiving have emerged to help mitigate the dual financial and caregiving burdens of inadequate access to home healthcare services [72,73]. Forgone employment contributed heavily to our composite measure of financial hardship in this study, though we did not analyze it as a separate variable. Future studies with a larger sample size may further delineate the associations between forgone employment and HN access in CMC.

The families of CMC frequently describe a fragile network of caregiving supports, in which instability of one support can have a profound impact on all others [74,75,76,77]. In addition to assessing the impact of median understaffed HN hours, we sought to evaluate how disruptions to home care services (as measured by the rate of acute care encounters in the six months prior to survey completion) related to family wellbeing. Total family impact scores and parent health-related quality of life scores were associated with the acute care rate, though in a bimodal distribution with the most positive scores (lowest family impact and highest health-related quality of life) being among participants within the middle tertile of the acute care rate. The reason for this relationship is not clear, though it raises questions about access to care. For instance, a low acute care rate may reflect health stability or, conversely, poor access to acute care when needed. It is also worth noting that, while HN availability is not an immediate concern while a child is hospitalized, in the US, home nurses are not paid while a child is admitted. Thus, a family may lose their assigned HN during a hospitalization and be unable to staff those hours after discharge. The relationship between acute care utilization and family wellbeing deserves further study. It is possible that unknown confounders may be responsible for this finding.

There were no associations between the proportion of staffed HN and survey measures of family wellbeing. This differs from findings in qualitative studies that have highlighted caregiver stress related to poor access to pediatric home healthcare [17,28,42,44,45,66]. This difference may be, in part, related to differences in study methodology; qualitative studies of HN use non-numeric data to describe experiences of HN care, rather than to quantify generalizable population patterns of HN delivery. It may also reflect the profound complexity of the home care landscape for CMC. HN is part of a complex patchwork of services and supports that families of CMC rely on for daily care [77,78,79]. Some of these supports are formal, such as HN, while others are informal, such as reliance on family members and friends for caregiving. This study measured receipt of HN but not of informal supports; it is unknown to what degree the families in this study relied on informal supports to meet caregiving needs for their CMC.

There are limitations to consider when interpreting our findings. Our sample was taken from a single children’s hospital in the US and all study participants spoke English and were legal guardians, which may have excluded foster families, non-legal carers, and those with limited English proficiency. Because of this, the present study is unable to assess the impact of language-based barriers or glean the perspectives of foster families. Self-reported race/ethnicity data were available for the child but not the parent, which may have limited the detection of associations with parent experiences of racism. Our data source did not include parental education data; incorporating parental education data in future studies will allow for a more nuanced understanding of the relationship between HN services and FFE. Participants were enrolled in the site’s multidisciplinary CMC clinic, which provided clinical service coordination support intended to improve HN staffing for CMC. As a result, this population may have higher HN staffing than other CMC. We did not account for care provided by patient care aides or certified nursing assistants (CNAs). These services do not require the same documentation as HN and, therefore, we were unable to track their contribution to home-based care. These services are not equivalent to HN, though we have observed clinically that families having difficulty staffing HN will instead utilize a CNA or patient care aide if available rather than forgo in-home services altogether [72,73]. The use of natural language processing, like any type of machine learning, carries risk of bias. We have attempted to mitigate that potential bias by validating a subset of the participant data with manual EHR review. We relied on EHR documentation to identify understaffed HN. This required a set of a priori assumptions to address non-numeric or incomplete documentation. We formulated these assumptions to be as conservative as possible, which may have resulted in over-representation of staffed HN. We did not have access to data regarding upstream leaks in the HN “leaky pipeline”, such as nonmedical variations in HN eligibility determination or hour allocation. Associations between understaffed HN and family wellbeing in this study reflect gaps in delivery of insurance-approved HN. There is no agreed upon outcome or instrument for measuring family wellbeing in CMC. The existing validated measures utilized in this study may not capture all the unique needs of this population. We were only able to identify acute care encounters occurring within our children’s hospital system. We expect uncaptured acute care encounters to be few since the study site is the only free-standing children’s hospital in the region and participants were enrolled in the CMC clinic that required residence near the hospital.

Future studies with a larger sample size and utilizing multiple sites may more precisely determine the impact of understaffed HN on family wellbeing. Notable geographic variation in approval of HN hours [14,15] may identify patterns of disparities in HN staffing beyond the shortage of available nurses. The development of home healthcare data-sharing consortia and development of consistent data entry practices will greatly improve the ability to evaluate HN delivery and impact on health outcomes. This study was conducted prior to the COVID-19 pandemic, which both exacerbated existing widespread HN shortages and brought about new opportunities for paid family caregiving; both of these phenomena deserve to be studied. Further study of home caregiving of CMC will provide a better understanding for how to bolster CMC health and family wellbeing.

## 5. Conclusions

The parents of the CMC in this study demonstrated a very high level of financial hardship and a significant association between receipt of less than half of approved HN hours and presence family financial hardship. Parental health-related quality of life and family impact were associated with acute care rate. Future multisite studies, including those using mixed methods approaches, are needed to better identify the relationship between understaffed HN and family wellbeing in this population. Future research should evaluate the impact of policies and programs that address financial and caregiving burden, such as paid family caregiving, as families navigate the reality of a persistent HN shortage. Improving access to and the coordination of home nursing services carries potential benefits to family wellbeing in addition to direct health benefits for CMC.

## Figures and Tables

**Table 1:** Participant characteristics.

Child Characteristics, n (%)		Participants
		n (%)
Total		72
Age, years	<1	6 (8.3%)
	1–2	14 (19.4%)
	3–5	20 (27.8%)
	6–11	20 (27.8%)
	12–17	12 (16.7%)
Sex	Male	43 (59.7%)
	Female	29 (40.3%)
Race/Ethnicity	Non-Hispanic White	48 (66.7%)
	Non-Hispanic Black	15 (20.8%)
	Non-Hispanic Other	6 (8.3%)
	Non-Hispanic Asian	2 (2.8%)
	Hispanic	1 (1.4%)
Insurance *	Public	42 (58.3%)
	Commercial	24 (33.3%)
	Both public and commercial	6 (8.3%)
Complex chronic conditions	2–3	15 (20.8%)
	4–5	32 (44.4%)
	≥6	25 (34.7%)
Approved HN hours per week	<40	13 (18.0%)
	40–80	39 (54.2%)
	>80	20 (27.8%)
Staffed HN per week	<50%	14 (19.4%)
	50–89%	14 (19.4%)
	90+%	44 (61.1%)
Acute Care Rate	Lowest Tertile [0.0, 5.6]	36 (50.7%)
	Middle Tertile [5.7, 11.4]	12 (16.9%)
	Highest Tertile [11.5, 135.1]	23 (32.4%)

* Insurance type reflects payor for acute care encounters, with one payor per encounter. HN: Home Nursing.

**Table 2:** Unadjusted and adjusted analyses of HN staffing and family wellbeing.

	Unadjusted		Adjusted	
	<50% Staffing	50–89% Staffing	<50% Staffing	50–89% Staffing
Parental Stress	−0.15 (−0.80, 0.48)	0.24 (−0.30, 0.82)	−0.24 (−0.96, 0.53)	0.25 (−0.29, 0.87)
Family Empowerment	0.04 (−0.58, 0.66)	−0.03 (−0.60, 0.52)	0.03 (−0.61, 0.72)	−0.03 (−0.65, 0.53)
Family Functioning	0.03 (−0.52, 0.62)	0.49 (−0.04, 1.04)	−0.04 (−0.69, 0.61)	0.48 (−0.08, 1.08)
Parental HRQL	−0.35 (−0.86, 0.13)	0.29 (−0.24, 0.82)	−0.36 (−0.90, 0.29)	0.32 (−0.22, 0.81)
Family Impact Total	−0.31 (−0.84, 0.22)	0.26 (−0.29, 0.81)	−0.36 (−0.93, 0.23)	0.28 (−0.28, 0.82)
Interpersonal Support	0.02 (−0.62, 0.64)	0.15 (−0.54, 0.79)	−0.03 (−0.69, 0.76)	0.11 (−0.51, 0.78)

Coefficients and bootstrapped confidence intervals are relative to the referent of ≥90% HN. Analyses adjusted for age, number of CCCs, and acute care rate. All *p* > 0.05.

**Table 3:** Adjusted HN staffing, acute care, and family financial hardship.

	Unadjusted Rate Ratio	Adjusted Rate Ratio
HN Staffing		
<50%	1.63 (1.19, 2.18)	1.51 (1.03, 2.16)
50–89%	1.07 (0.59, 1.64)	1.08 (0.63, 1.68)
90+%	-ref-	-ref-
Acute care		
Lowest tertile	-ref-	-ref-
Middle tertile	1.24 (0.82, 1.86)	1.16 (0.78, 1.60)
Highest tertile	1.29 (0.93, 1.78)	1.18 (0.82, 1.45)

Analyses adjusted for age, number of CCCs, acute care rate, and percentage of HN staffing.

**Table 4:** Unadjusted and adjusted analyses of acute care rate and family wellbeing.

	Unadjusted		Adjusted	
	Middle Tertile	Highest Tertile	Middle Tertile	Highest Tertile
Parental Stress	0.32 (−0.42, 1.04)	0.18 (−0.32, 0.67)	0.39 (−0.45, 1.20)	0.23 (−0.31, 0.78)
Family Empowerment	0.03 (−0.63, 0.66)	0.01 (−0.50, 0.53)	0.02 (−0.72, 0.72)	0.00 (−0.61, 0.55)
Family Functioning	0.46 (−0.18, 1.08)	0.04 (−0.49, 0.56)	0.53 (−0.13, 1.25)	0.10 (−0.44, 0.65)
Parental HRQL	0.70 (0.17, 1.20)	−0.17 (−0.69, 0.36)	0.81 (0.27, 1.32)	−0.12 (−0.67, 0.45)
Family Impact Total	0.67 (0.10, 1.21)	−0.05 (−0.56, 0.46)	0.77 (0.24, 1.30)	0.01 (−0.56, 0.61)
Interpersonal Support	0.26 (−0.39, 0.85)	−0.02 (−0.57, 0.52)	0.26 (−0.37, 0.90)	0.03 (−0.61, 0.64)

Coefficients are relative to referent of lowest tertile (lowest acute care rate). Analyses adjusted for age, number of CCCs, and percentage of HN staffing.

## Data Availability

The datasets presented in this article are not readily available because the data are part of an ongoing study.

## Supplementary Materials

The following supporting information can be downloaded at: https://www.mdpi.com/article/10.3390/children12040455/s1, Table S1: Medical Record Query Strategy and A Priori Assumptions for HN Staffing Documentation; Table S2: Unadjusted Composite Count of Family Financial Hardship.
