# Supplementary material for: Understaffed Home Nursing and Wellbeing of Families of Children with Medical Complexity
Source: Children (Basel). 2025 Mar 31;12(4):455. doi: 10.3390/children12040455 (PMC12025880; doi:10.3390/children12040455)
Supplement: Supplementary file 1 [file children-12-00455-s001.zip › children-3499590-supplementary.pdf]

children

MDPI

Supplementary Materials
Understaffed Home Nursing and Wellbeing of Families of Children with Medical Complexity

Supplementary Table S1. Medical Record Query Strategy and A Priori Assumptions for HN Staffing Documentation

**Natural Language Processing Query Strategy**	
Search terms	"PDN", "private duty nurse", "private duty nursing", "home nursing", "home nurse", "home health", "home RN", "home LPN", "home CA", "nursing agency", "PCA", "patient care assistant", "skilled caregiver", "paid caregiver", "consumer directed services", "current private duty agency", "private duty instructions"
Query definitions	• 50 characters before and 100 characters after each of these terms;<br>• 200 characters after the term "current private duty agency";<br>• If a number occurs in the 100 characters after a search term, add an additional 100 characters after that number;<br>• If the terms "cut down" or "decrease(d)" or "increase(d)" occur within the 50 characters before or 100 characters after a search term, add another 300 characters after the term;<br>• Manual full chart review utilized if additional documentation context required to determine HN hour staffing.
***A Priori* Assumptions for Inconsistent or Non-Numeric HN Documentation**	
- Hours/week as standard unit for HN coverage, convert other units to this for the purpose of comparison.<br>- One "shift" ("night coverage", "day coverage", days per week without hours noted) = 8 hours, unless otherwise noted.<br>- One unit = 15 minutes.<br>- One month = 30 days = 4.29 weeks.<br>- If no documentation of understaffed HN, assume full HN staffing per Home Health Plan of Care.<br>- If clinically documented HN hours less than in Home Health Plan of Care, the clinically documented number represents staffed HN hours.<br>- If clinical documentation of HN hours without comment about understaffing, and there is no documented Home Health Plan of Care covering that date range, the clinically documented number represents approved HN hours (implying 100% staffed).<br>- The date of documentation will be assumed to be the date of change in hours staffed or approved, unless an alternate date (or dated event, such as hospital discharge) of the service change is documented.<br>- If there are documentation gaps (e.g., no Home Health Plan of Care documents covering a certification period), assume that number of approved and staffed HN hours remain unchanged during that time.<br>- HN and PCA services are qualitatively different, but some families seek PCA hours when no HN hours are available. PCA are not considered a replacement for HN and are not included when documenting either approved or staffed hours for this study.	

- If clinical documentation notes that HN is “staffed inconsistently” or “staffed sporadically” but no numeric details, these dates are documented as fully staffed. .

Supplementary Table S2. Unadjusted Composite Count of Family Financial Hardship

Composite Score	Forgone Family Employment	Food Insecurity	Housing Insecurity	Energy Insecurity	Frequency
0	0	0	0	0	8
1	0	0	0	1	4
1	1	0	0	0	32
2	1	0	0	1	12
2	1	0	1	0	6
3	1	0	1	1	6
3	1	1	0	1	1
3	1	1	1	0	1
4	1	1	1	1	2

* The four binary indicators for financial hardship and were summed to create a composite score.
